# The Synthesis and Characterization of Hydroxyapatite-*β*-Alanine Modified by Grafting Polymerization of *γ*-Benzyl-l-glutamate-N-carboxyanhydride

**DOI:** 10.3390/molecules181113979

**Published:** 2013-11-13

**Authors:** Yukai Shan, Yuyue Qin, Yongming Chuan, Hongli Li, Minglong Yuan

**Affiliations:** 1 Engineering Research Center of Biopolymer Functional Materials of Yunnan, Yunnan University of Nationalities, Kunming 650500, China; 2 Institute of Chemical Engineering, Kunming University of Science and Technology, Kunming 650500, China

**Keywords:** hydroxyapatite, *β*-alanine, *γ*-benzyl-l-glutamate-N-carboxyanhydride, surface modification, hydroxyapatite-*β*-alanine-poly(*γ*-benzyl-l-glutamates)

## Abstract

In this study, hydroxyapatite (HAP) was surface-modified by the addition of *β*-alanine (*β*-Ala), and the ring-opening polymerization of *γ*-benzyl-l-glutamate-N-carboxy-anhydride (BLG-NCA) was subsequently initiated. HAP containing surface poly-*γ*-benzyl-l-glutamates (PBLG) was successfully prepared in this way. With the increase of PBLG content in HAP-PBLG, the solubility of HAP-PBLG increased gradually and it was ultimately soluble in chloroform. HAP-PLGA with surface carboxyl groups was obtained by the catalytic hydrogenation of HAP-PBLG. In the process of HAP modification, the morphology changes from rod to sheet and from flake to needle. The effect of BLG-NCA concentration on the character of hydroxyapatite-*β*-alanine-poly(*γ*-benzyl-l-glutamate) (HAP-PBLG) was investigated. The existence of amino acids on the HAP surfaces was confirmed in the resulting Fourier transform infrared (FTIR) spectra. The resulting powder X-ray diffraction patterns indicated that the crystallinity of HAP decreased when the ratio of BLG-NCA/HAP-NH_2_ increased to 20/1. Transmission electron microscopy (TEM) indicated that the particle size of HAP-PBLG decreased significantly and that the resulting particles appeared less agglomerated relative to that of the HAP-NH_2_ crystals. Furthermore, ^1^H-NMR spectra and FTIR spectra revealed that hydroxyapatite-*β*-alanine-poly (l-glutamic acid) (HAP-PLGA) was able to successfully bear carboxylic acid groups on its side chains.

## 1. Introduction

For many years, there has been a growing interest in the biomedical field in preparing and applying hydroxyapatite (HAP, [Ca_10_(PO_4_)_6_(OH)_2_]) because it has good biocompatibility and it is the main inorganic constituent of animal hard tissues, such as bone and teeth [1,2]. As a type of functional material, nanometre-size HAP has some defects: it is brittle and lacks functional groups on its surface, and the stability of the colloids in the process of the formation of nanocrystals is weak [3]. The goal of materials chemistry is to develop materials with desired properties. HAP is usually synthesized by the hydrothermal method, without modification, so the surface functional groups are hydroxyl groups [4]. In order to meet the needs of practical applications, it is necessary to tailor the chemical nature of the HAP nanosurface. It has been reported that the functional groups, pH, and charge on the surface of HAP have great effects on its surface properties [5,6]. Therefore, the modification of surface functional groups on the HAP is an effective method to obtain the unique HAP properties.

The literature reports some methods to overcome the brittleness of HAP and modify the surface functional groups, such as by grafting a biodegradable polymer onto the surface of HAP *etc.* Zreiqat [6] developed a composite biphasic calcium phosphate (BCP) scaffold by coating a nanocomposite layer, consisting of hydroxyapatite (HAP) nanoparticles and polycaprolactone (PCL), over the surface of BCP. Lee [7] described a rational approach to hydroxyapatite (HAP) nanosurface modification for the graft polymerization of caprolactone and lactide. The research showed that grafted PCL on HAP surface had enhanced the colloidal stability, and the HAP could be well dispersed in organic solvents.

It is well-known that poly(amino acids)s are biodegradable and biocompatible polymers, especially poly(amino acids)s containing carboxylate groups. They were specifically found to inhibit the growth rate of hydroxyapatite when present in solution [8,9]. Amino acids also have been used *in vitro* to synthesise HAP-macromolecule composites with reduced crystal dimensions for hard tissue replacement [10,11,12,13]. The incorporation of amino acids controlled the crystal dimensions and favours osteoblast proliferation, the activation of osteoblast metabolism and differentiation, which are all of high importance for potential biomedical applications. 

Many studies have paid attention to the synthesis of inorganic-organic nanocomposites based on the grafting of organic polymers onto the surface of HAP. The resulting materials might display the performance characteristics of both molecules and complement each other. Modifications of HAP by polychitosan, polyethylene oxide, and PMMA have been reported [14,15,16], although the methods of HAP modification were complex.

Many literature studies of modified HAPs were mainly focused on the surface modification agent and the changes that occurred in the HAP morphology, the effects of nanometre size, the surface functional groups on the grafted HAP, and improvements in the hydrophilicity of HAP, *etc.* [17,18,19]. However, few studies have been published with the goal of modifying HAP to improve its solubility in organic solvents.

In this study, we used poly (amino acids)s to modify HAP and to prepare hydroxyapatite-*β*-alanine-poly(*γ*-benzyl-l-glutamates) (HAP-PBLG) nanocrystals that possessed an architecture similar to that of natural bones, which was found to be more bioactive when compared to non-substituted HAP. In the case of amino acids with –COOH functional groups, this modification would offer the possibility of producing multifunctional HAPs that consist of hydrophilic/hydrophobic, soft/hard chain segments, and functional groups, and thus constitutes an attractive means of modulating the basic properties of each material, especially to improve the solubility of HAP in organic solvents.

## 2. Results and Discussion

### 2.1. Grafting Polymerization of BLG-NCA on HAP-Ala Crystals

The amino-functionalised HAP (HAP-NH_2_) was obtained by the strong coordination of the carboxyl groups of alanine to the Ca^2+^ ions of HAP. The structure of HAP-NH_2_ was characterised by elemental analysis and TEM and proved to be consistent with that given in [20]. HAP-PBLG was obtained by the ring-opening polymerization of BLG-NCA, initiated by the amino-functionalised HAP. The results are shown in Table 1. 

**Table 1:** The yield, Mn, PDI, percentage grafting, and Ca content of HAP-PBLG.

Sample ID	Yield (%)	Mn ^a^	PDI ^a^	Percentage grafting (%) ^b^	Ca content (%) ^c^	
					Theoretical value	Measured value
HAP-NH_2_	--	--	--	--	38.46	15.89
HAP-5	54.4	--	--	39.31	2.65	4.03
HAP-10	60.6	--	--	66.36	1.45	2.56
HAP-20	61.8	--	--	70.03	0.76	1.10
HAP-50	85.0	148,000	1.01	76.79	0.31	0.44
HAP-100	70.8	142,000	1.02	83.18	0.16	0.23
HAP-200	81.8	131,000	1.02	80.37	0.08	0.23
HAP-300	70.2	153,000	1.03	78.15	0.05	0.33

^a^ Determined by GPC; ^b^ Determined by TGA; ^c^ Determined by a flame atomic absorption spectrophotometry.

As seen from the Table, the BLG-NCA/HAP-NH_2_ ratio (B/H) could affect the molecular weight and yield of HAP-PBLG. With the increase of B/H, the yield showed a slight increase; the molecular weight also increased with increasing B/H. This was might be due to the higher concentration of the HAP initiator, which increased the number of active species, leading to the low polymerisation degree of the BLG-NCA monomer. When the B/H ratio was in the range from 5–20, the molecular weight of HAP-PBLG could not be detected by GPC due to its insolubility in the selected solvent. HAP was not soluble in any typical organic solvents such as chloroform, dimethylformamide (DMF), or dimethylsulfoxide (DMSO). A study of the solubility of HAP-PBLG showed that, along with the increase of the polymer ratio, the solubility of HAP in organic solvents had been significantly improved, with solutions changing from a white emulsion to a homogeneous solution. When the B/H ratio was in the range from 50 to 300, the molecular weight of HAP-PBLG could now be detected by GPC. The GPC analysis results showed that the molecular weight distribution of HAP-PBLG was rather narrow. In our study, the removal of the benzyl group from HAP-PBLG was conducted by catalytic hydrogenation, resulting in the introduction of carboxyl functional groups on the surface of HAP.

**Figure 1:**
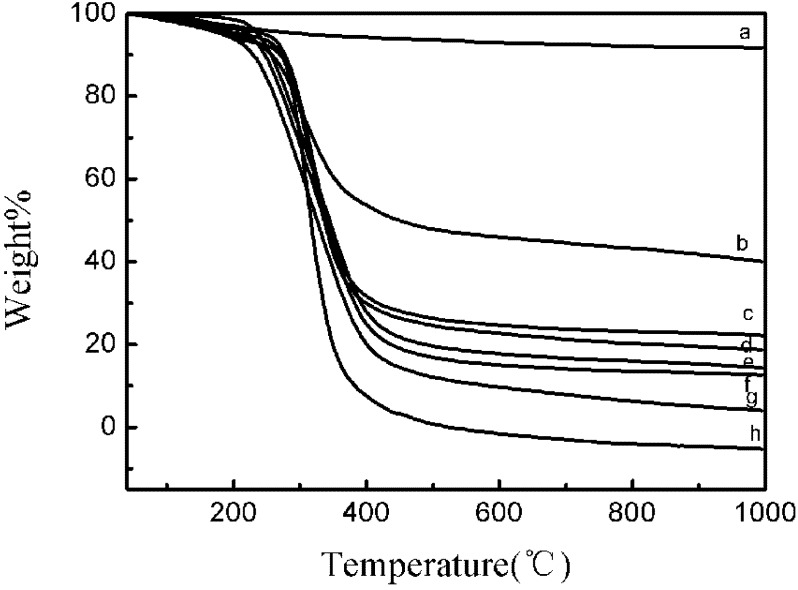
TGA curves of (a) HAP-NH_2_, (b) HAP-5, (c) HAP-10, (d) HAP-20, (e) HAP-50, (f) HAP-300, (g) HAP-200, and (h) HAP-100.

The amount of grafted PBLG on the HAP-NH_2_ crystals was estimated by TGA. As shown in Figure 1, the HAP-NH_2_ crystals displayed a weight loss of less than 6% up to 1,000°C, which was caused by the release of absorbed water [4]. The weight loss of the HAP-NH_2_ crystals was lower than that of the HAP-PBLG crystals (HAP-5, HAP-10, HAP-20, HAP-50, HAP-100, HAP-200, and HAP-300), fabricated by the grafting polymerisation of PBLG onto the HAP-NH_2_ surface. The weight loss of the HAP-PBLG crystals increased when the feed molar ratio of BLG-NCA/HAP-NH_2_ was decreased from 5/1 to 100/1. 

The percentage grafting rate and Ca content of the surface modified HAP-PBLG are shown in Table 1. As could be seen from this data, the feed weight ratio of BLG-NCA/HAP-NH_2_ was varied to control the degree of grafting polymerization of PBLG on the HAP-NH_2_ crystals. The percentage grafting rate increased significantly with the increase of BLG-NCA. The highest percentage grafting rate was observed when the feed molar ratio of BLG-NCA/HAP-NH_2_ reached 100/1. This value was approximately 111% higher than that of the BLG-NCA/ HAP-NH_2_ crystals with a feed molar ratio of 5/1. The content of Ca ions of HAP-PBLG was lower than that of the HAP-NH_2_ crystals and decreased significantly with the increase of BLG-NCA.

### 2.2. The Synthesis of Hydroxyapatite-β-alanine-poly (l-glutamic acid) (HAP-PLGA)

HAP-PLGA could be synthesised by the hydrogenation of HAP-PBLG in the presence of Pd/C; using this method, it was possible to introduce carboxyl groups onto the surface of HAP. The presence of these carboxyl groups on the surface of HAP could improve its hydrophilicity. Other active drug molecules could be introduced into the surface of HAP through the carboxyl groups.

### 2.3. XRD Patterns

**Figure 2:**
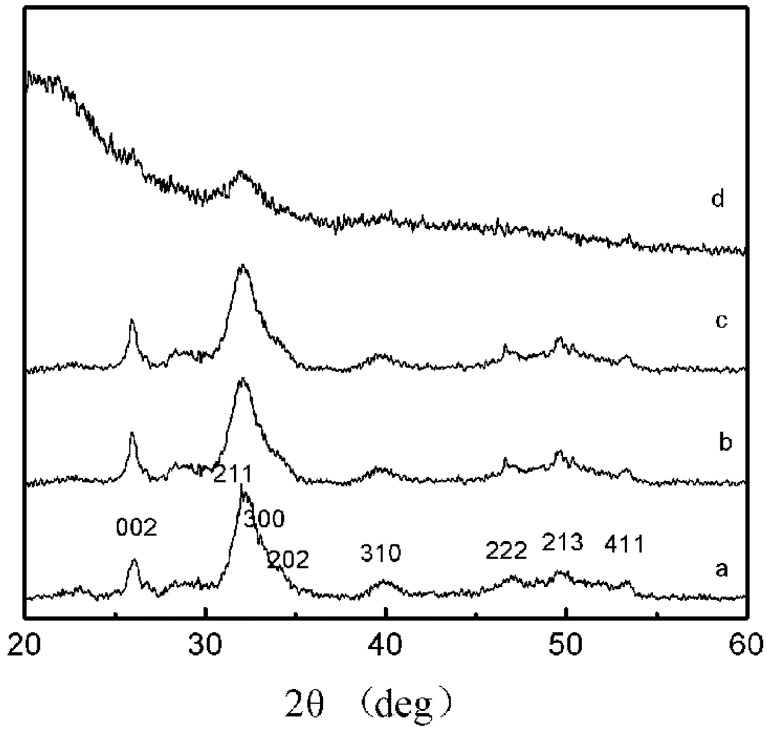
Powder X-ray diffraction patterns of (**a**) HAP, (**b**) HAP-NH_2_, (**c**) HAP-5, and (**d**) HAP-20.

From Figure 2, it could be observed that the HAP powder presented wide diffraction peaks, which were due to the low degree of crystallinity induced in the sample [20]. The powder X-ray diffraction patterns of HAP, HAP-NH_2_, HAP-5, and HAP-20 were shown in Figure 2. The XRD analysis indicated that the characteristic peaks of 2 samples, HAP- NH_2_ and HAP-PBLG (5/1) (Figure 2b,c), at 002, 211, 300, 202, 310, 222, 213, 411, agreed with the standard spectra of HAP (Figure 2a). The patterns of HAP, HAP-NH_2_, and HAP-PBLG (5/1) showed that these samples were composed of HAP as a unique crystalline phase. The formation of a secondary phase due to chemical reaction could not be detected. Furthermore, the amino acids provoked a broadening of the diffraction peaks, which increased with increasing content of PBLG. When the feed molar ratio of BLG-NCA/HAP-NH_2_ was increased to 20/1, the diffraction peak broadening was accompanied by a decrease in peak intensity; this phenomenon was clear evidence of a decreasing trend in crystallinity [21]. According to Figure 2a, c and d, it could be seen that the modification of HAP with PBLG did not change the crystal structure of the HAP. However, as the content of PBLG in the HAP increased, the crystallinity of HAP decreased. This also implied that the PBLG was successfully grafted onto the HAP.

### 2.4. TEM

**Figure 3:**
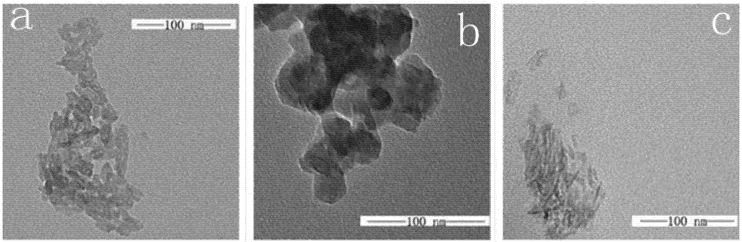
TEM images of (**a**) HAP, (**b**) HAP-NH_2_, and (**c**) HAP-5.

The TEM micrographs presented in Figure 3 showed the effects of *β-*Ala and PBLG on the morphology of the HAP crystals. It had been reported that HAP could adopt a variety of morphologies, including the common rod-like, flake and acicular forms [17,22,23]. From Figure 3, it could be seen that unmodified HAP existed in a rod shape (Figure 3a). However, when using the *β-*alanine-modified HAP, HAP-NH_2_ was visualised as a sheet (Figure 3b), and when further modified by PBLG, HPA-PBLG presented an acicular morphology (Figure 3c). Compared with the former, the dispersion of the HAP was significantly improved. Pure HAP crystals were unable to maintain colloidal stability and precipitated very quickly (Figure 3a). The morphology of the hydroxyapatite powder synthesised in the presence of *β*-Ala revealed a highly agglomerated apatite powder (Figure 3b). The particle size increased from 15.3 nm (HAP) to 27.5 nm (HAP-NH_2_). After the HAP-Ala was modified by PBLG (BLG-NCA/HAP-NH_2_ = 5/1) using grafting polymerization, HAP-5 powder with well-defined needle-like structures was obtained by a significant reduction in the particle size (Figure 3c). The particle size of HAP-5 significantly decreased to 9.1 nm, and the resulting particles appeared less agglomerated. Compared with pure HAP and HAP-NH_2_ particles, the needle-like nanocrystals (HAP-5) possessed greater similarity to the morphology and crystal structure of natural apatite [24]. Adsorption on the surface of the HAP particles enhances the colloidal stability through interparticle electrostatic repulsion [25].

### 2.5. ^1^H-NMR Spectra

It is well known that HAP is not soluble in organic solvents such as chloroform, DMSO and DMF. To determine whether the surface of HAP has been successfully been modified, usually NMR is used for structural analysis, but because HAP is insoluble in organic solvents, only solid NMR can be used for this analysis [26,27]. In our study, we found that, when the content of PBLG on the HAP surface reached a certain proportion, such as HAP-50, the HAP could be dissolved in chloroform, DMF, and other organic solvents, which could be used to identify its structure through magnetic analysis by dissolving the HAP in an organic solvent. 

**Figure 4:**
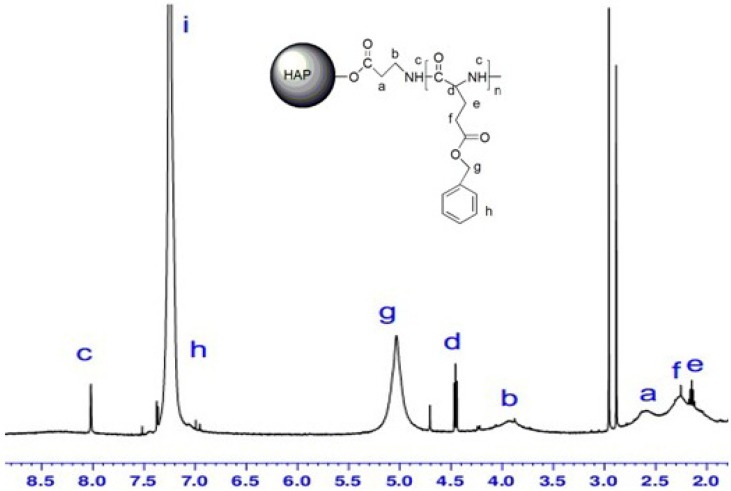
^1^H-NMR spectrum of HAP-100 in CDCl_3_.

Figure 4 shows the ^1^H-NMR spectrum of HAP-100 in CDCl_3_. The peaks at 2.5–2.7 and 3.8–4.0 ppm were assigned to the methylene protons of the *β*-alanine unit, and the peaks at 5.0, 7.3, and 8.0 ppm were attributed to the methylene, aromatic, and amino protons of the polymerization of the PBLG unit [28]. The nuclear magnetic resonance analysis results showed that the PBLG had been successfully grafted onto the HAP surface and could exist stably in chloroform. 

**Figure 5:**
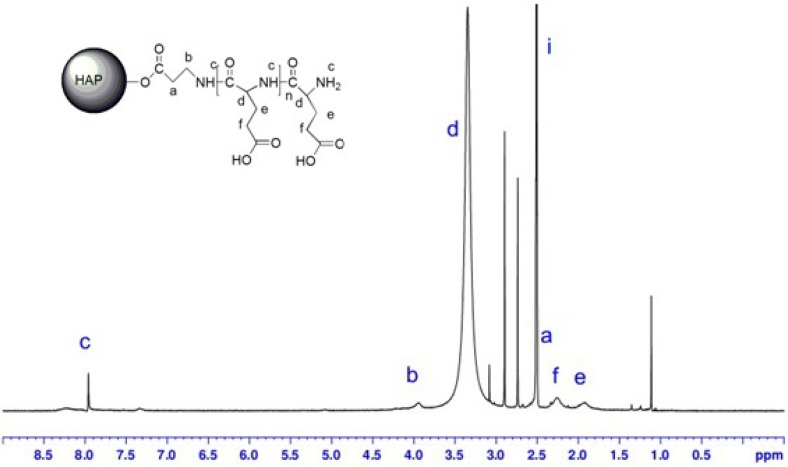
^1^H-NMR spectrum of HAP-100-PLGA in DMSO.

Figure 5 shows the ^1^H-NMR spectrum of HAP-PLGA in DMSO. The disappearance of the peak at 5.0 ppm confirmed that the benzyl group was missing. This demonstrated that the carboxyl group had been successfully grafted onto the surface of the HAP.

### 2.6. FTIR Spectra

**Figure 6:**
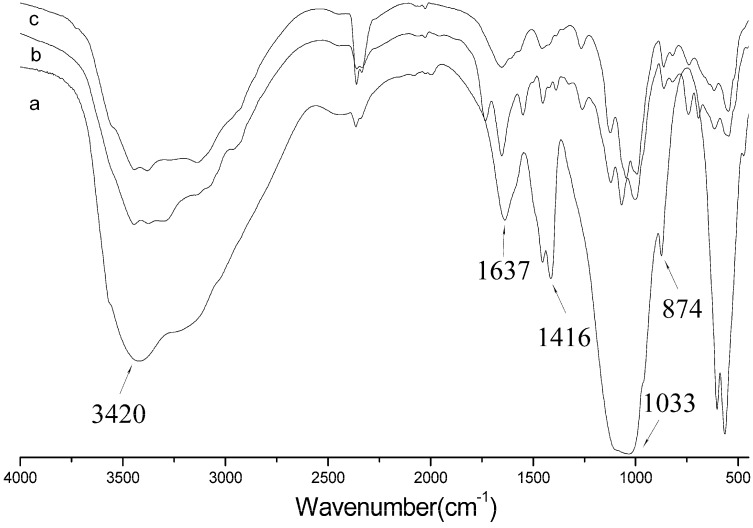
FTIR spectra of (a) HAP-NH_2_; (b) HAP-100 and (c) HAP-100-PLGA.

Figure 6 shows the FTIR spectra of HAP-NH_2_, HAP-100, and HAP-PLGA. FTIR analysis suggested that the carboxylic groups of the amino acids interacted with the calcium ions of HAP. The characteristic bands for PO_4_^3−^ appeared at 874 cm^−1^ and 1,033 cm^−1^; the characteristic bands for C=O appeared at 1,637 cm^−1^; and the characteristic bands for N-H appeared at 3,420 cm^−1^. The bands at 1,416 cm^−1^ were ascribed to CO_3_^2−^ bands [10].

The relative intensities of OH^−^ stretching decreased as a function of the amino acid content [12]. Figure 6b shows further absorption bands in the range from 2,000 to 1,200 cm^−^^1^. Furthermore, PBLG grafting led to the presence of new peaks at 1,591 cm^−^^1^ (Figure 6b). This belonged to the C–O stretching of PBLG on the surface of the HAP-NH_2_ crystals. The phenyl peaks appeared at 1,652, 1,549, and 1,452 cm^−1^. The characteristic N–H bands shifted to 3,385 cm^−1^. Hence, the existence of PBLG on the HAP-NH_2_ surfaces was confirmed.

As expected, the intensity of the characteristic bands for phenyl flattened (Figure 6c). The characteristic bands for –OH appeared at 3,450 cm^−1^. This showed that HAP-PLGA could successfully support the presence of carboxylic acid groups. Similarly modified HAP had good biocompatibility and had been applied in the biomedical field. Other researchers have used modified HAP as a drug carrier and bone repair material [29,30]. HAP with carboxyl groups on the surface could be modulated through the carboxyl group activity, so these groups could be used for further connections with other drug molecules and widen the range of applications of HAP in the biomedical field.

## 3. Experimental

### 3.1. Materials

Ca(OH)_2_ was purchased from Fengchuan Chemical Instrument Co., Ltd. (Tianjin, China). H_3_PO_4_ was purchased from Tianjin Damao Chemical Instrument Co., Ltd. (Tianjin, China). *β*-alanine and *γ*-benzyl-l-glutamate were obtained from Sigma-Aldrich Co. (Shanghai, China) and were of analytical purity. All solutions were prepared with deionised water.

### 3.2. Synthesis of HAP-PBLG

**Scheme 1:**
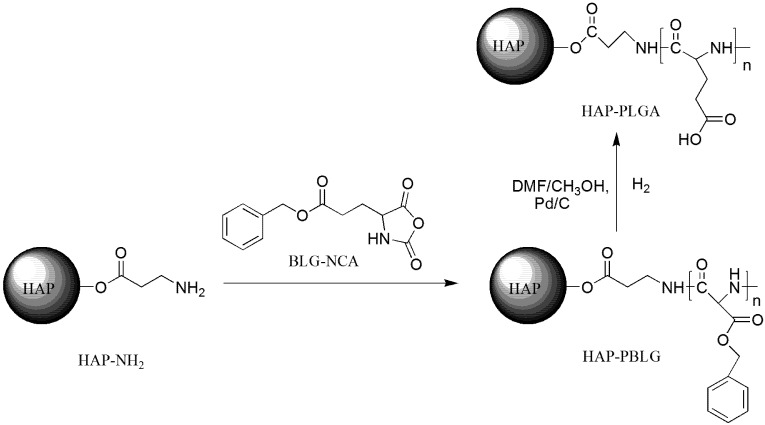
An illustration of the procedure used to prepare HAP-PLGA.

The procedure for preparing HAP- PBLG-NCA was shown in Scheme 1. The calcium solution was prepared by dissolving Ca(OH)_2_ (0.1 mol) in deionised water (5,000 mL), and stirring for 24 h at 25 °C. Then, H_3_PO_4_ (0.06 mol) and *β*-alanine (*β*-Ala, 0.1 mol) were added, respectively. The calcium solution was continuously stirred for an additional 24 h at 25 °C. The solution was allowed to stand to obtain a white precipitate. The white precipitate was collected by centrifugation and then was washed with deionised water three times, followed by drying in vacuum for 48 h to give hydroxyapatite-*β*-alanine (HAP-NH_2_) crystals [31].

*γ*-Benzyl-l-glutamate-*N*-carboxyanhydride (BLG-NCA) was synthesised according to the method of Mori *et al.* [32] For the grafting polymerisation of BLG-NCA on the HAP-NH_2_ crystals, the BLG-NCA solution was prepared by dissolving BLG-NCA in dimethylformamide (DMF), and the BLG-NCA solution was subsequently mixed with various amounts of HAP-NH_2_ (Table 2). The content was continuously stirred for 48 h at room temperature under a N_2_ atmosphere. Alcohol was added to the formed suspension to yield a white precipitate. The white precipitate was washed and dried to obtain hydroxyapatite-*β*-alanine-poly(*γ*-benzyl-l-glutamates) (HAP-PBLG).

**Table 2:** Samples synthesized with different molar ratios of BLG-NCA and HAP-NH_2_.

Sample ID	BLG-NCA/HAP-NH_2_
HAP-5	5/1
HAP-10	10/1
HAP-20	20/1
HAP-50	50/1
HAP-100	100/1
HAP-200	200/1
HAP-300	300/1

To deprotect the HAP-PBLG, HAP-PBLG (HAP-100, 0.5 g) was dissolved in a solution of DMF (45 mL) and methanol (5 mL). Then, palladium-carbon catalyst (2.5 g, 5% wt.) was added. The resulting solution was continuously stirred for 18 h. *tert*-Butylmonomethyl ether was added to obtain a precipitate. The precipitate was collected, washed, and dried to obtain hydroxyapatite-*β*-alanine-poly(l-glutamic acid) (HAP-PLGA).

### 3.3. Characterization

The amount of surface-grafted polymer on the HAP was determined using thermal gravimetric analysis (TGA; STA 449, Netzscn, Germany). TGA measurements were carried out from 40 °C to 1,000 °C at a rate of 10 °C min^−1^ under a nitrogen atmosphere on approximately 10 mg of material. The amount of surface-grafted polymer was determined as the percentage weight loss during heating.

The chemical composition of HAP-PBLG was measured by dissolving each sample (100 mg) in 0.1 N HNO_3_ solutions (5 mL). The calcium concentrations of the samples were determined by flame atomic absorption spectrophotometry (AA-6300, Shimadzu, Kyoto, Japan).

The molecular weight and molecular distribution were determined by GPC using a Waters Associates (Milford, MA, USA) model ALC/GPC 244 apparatus, at 40 °C, with a refractometer as the detector, DMF as the solvent, and calibration with polystyrene standards. Four specimens were tested under each condition.

To identify the obtained precipitates (HAP-PBLG), X-ray diffraction analysis was performed with an X-ray powder diffractometer (Rigaku D/max 2200, Tokyo, Japan). The data were collected over the 2θ range 20°–60° with a step size of 0.05°. 

To examine the colloidal stability, the samples were dispersed in distilled water under ultrasonic treatment for 2 min and then were dropped onto carbon-coated copper grids for observation by transmission electron microscopy (TEM) using a JEM-2100 (JEOL, Tokyo, Japan), operating at an acceleration voltage of 200 kV. The sample aspect ratio was calculated from the average length and width of 30 grains randomly selected in the TEM images.

Fourier transform infrared (FTIR) spectra were recorded on pressed KBr pellets unsing a iS10 FTIR spectrometer (Nicolet Nexus spectrometer, Franklin, MA, USA) at room temperature in the range between 4,000 and 400 cm^−1^ with a resolution of 4 cm^−1^, and each spectrum was the average of 32 scans.

^1^H-NMR spectra of HAP-100 were recorded with a Bruker-400 spectrometer (Bruker, Zurich, Switzerland) at room temperature, using CDCl_3_ as the solvent, while ^1^H-NMR spectra of HAP-100-PLGA were recorded with DMSO as the solvent.

## 4. Conclusions

In this study, HAP was surface-modified by the addition of *β*-alanine (*β*-Ala), followed by the ring-opening polymerization of BLG-NCA. A novel inorganic-organic nanocomposite was successfully prepared. In the process of HAP modification, the morphology was altered from rod to sheet and subsequently changed from flake to needle. The primary amino-terminated HAP proved to be an effective initiator for the ring-opening polymerisation of amino acid N-carboxyanhydrides. HAP containing poly(*γ*-benzyl-l-glutamates) (PBLG) on its surface was successfully prepared in this way. With the increase of the PBLG content of HAP-PBLG, the solubility of HAP-PBLG in organic solvents increased gradually. This form could even be dissolved in chloroform. HAP-PLGA with surface carboxyl groups could be obtained by the catalytic hydrogenation of HAP-PBLG. 

## References

[1] Bouyer E., Gitzhofer F., Boulos M.I. (2000). Morphological study of hydroxyapatite nanocrystal suspension. J. Mater. Sci. Mater. Med..

[2] Pielichowsa K., Blazewicz S. (2010). Bioactive polymer/hydroxyapatite (nano) composites for bone tissue regeneration. Adv. Polym. Sci..

[3] Wang S.G., Wen S.H., Shen M.W., Guo R., Cao X.Y., Wang J.H., Shi X.Y. (2011). Aminopropyltriethoxysilane-mediated surface functionalization of hydroxyapatite nanoparticles: Synthesis, characterization, and *in vitro* toxicity assay. Nanomedicine.

[4] Lee S.C., Choi H.W., Lee H.J., Kim K.J., Chang J.H., Kim S.Y., Choi J., Oh K., Jeong Y.K. (2007). *In-situ* synthesis of reactive hydroxyapatite nano-crystals for a novel approach of surface grafting polymerization. J. Mater. Chem..

[5] Ethirajan A., Ziener U., Landfester K. (2009). Surface-functionalized polymeric nanoparticles as templates for biomimetic mineralization of hydroxyapatite. Chem. Mater..

[6] Roohani S.R., Nouri S.N., Lu Z.F., Appleyard R., Zreiqat H. (2010). The influence hydroxyapatite nanoparticle shape and size on the properties of biphasic calcium phosphate scaffolds coated with hydroxyapatite–PCL composites. Biomaterials.

[7] Lee H.J., Choi H.W., Kim K.J., Lee S.C. (2006). Modification of hydroxyapatite nanosurfaces for enhanced colloidal stability and improved interfacial adhesion in nanocomposites. Chem. Mater..

[8] Verma D., Katti K.S., Katti D.R. (2008). Effect of biopolymers on structure of hydroxyapatite and interfacial interactions in biomimetically synthesized hydroxyapatite/biopolymer nanocomposites. Ann. Biomed. Eng..

[9] Moreno E.C., Kresak M., Hay I. (1984). Adsorption of molecules of biological interest onto hydroxyapatite. Calcif. Tissue Int..

[10] Zhang G., Chen J.D., Yang S., Yu Q.F., Wang Z.L., Zhang Q.P. (2011). Preparation of amino-acid-regulated hydroxyapatite particles by hydrothermal method. Mater. Lett..

[11] Boanini E., Torricelli P., Gazzano M., Giardino R. (2006). Nanocomposites of hydroxyapatite with aspartic acid and glutamic acid and their interaction with osteoblast-like cells. Biomaterials.

[12] Sakuragi M., Kitajima T., Nagmune T., Ito Y. (2011). Recombinant hBMP4 incorporated with non-canonical amino acid for binding to hydroxyapatite. Biotechnol. Lett..

[13] Weiger M.C., Park J.J., Doy M.D., Stafford C.M., Karim A., Becker M.L. (2010). Quantification of the binding affinity of a specific hydroxyapatite binding peptide. Biomaterials.

[14] Kim S.E., Choi H.W., Lee H.J., Chang H.J., Choi J., Kim K.J., Lim H.J., Junc Y.J., Lee S.C. (2008). Designing a highly bioactive 3D bone-regenerative scaffold by surface immobilization of nano-hydroxyapatite. J. Mater. Chem..

[15] Murugan R., Ramakrishna S. (2004). Coupling of therapeutic molecules onto surface modified coralline hydroxyapatite. Biomaterials.

[16] Liu Q., de Wijn J.R., de Groot K., van Blitterswijk C.A. (1998). Surface modification of nano-apatite by grafting organic polymer. Biomaterials.

[17] Yao X., Yao H.W., Li G.Y., Li Y.T. (2010). Biomimetic synthesis of needle-like nano-hydroxyapatite templated by double-hydrophilic block copolymer. J. Mater. Sci..

[18] Okabe Y., Kurihara S., Yajima T., Seki Y., Nakamura I., Takanob I. (2005). Formation of super-hydrophilic surface of hydroxyapatite by ion implantation and plasma treatment. Surf. Coating. Tech..

[19] Taguchi T., Muraoka Y., Matsuyama H., Kishida A., Akashi M. (2000). Apatite coating on hydrophilic polymer-grafted poly(ethylene) films using an alternate soaking process. Biomaterials.

[20] Li C.F., Ge X.L., Liu S.G., Li G.C., Zhang A.J., Bai J.H., Su C.H., Ding R. (2011). Redispersible dried hydroxyapatite particles with grafted pH-sensitivity polymer brushes of poly(styrene-co-4-vinylpyridine). Powder Technol..

[21] Gopi D., Nithiya S., Kavitha L., Ferreira J.M.F. (2012). Amino acid-assisted synthesis of strontium hydroxyapatite bone cement by a soft solution freezing method. Bull. Mater. Sci..

[22] Vuk U., Dragan P.U. (2011). Nanosized hydroxyapatite and other calcium phosphates: Chemistry of formation and application as drug and gene delivery agents. J. Biomed. Mater. Res. Part B Appl. Biomater..

[23] Du X.W., Chu Y., Xing S.X., Dong L.H. (2009). Hydrothermal synthesis of calcium hydroxyapatite nanorods in the presence of PVP. J. Mater. Sci..

[24] Hao J.Y., Liu Y., Zhou S.B., Li Z., Deng X.M. (2003). Investigation of nanocomposites based on semi-interpenetrating network of [l-poly (*ε*-caprolactone)]/[net-poly (*ε*-caprolactone)] and hydroxyapatite nanocrystals. Biomaterials.

[25] Matsumoto T., Okazaki M., Inoue M., Hamada Y., Taira M., Takahashi J. (2002). Crystallinity and solubility characteristics of hydroxyapatite adsorbed amino acid. Biomaterials.

[26] Turki T., Aissaa A., Bacb C.G., Rachdi F., Debbabi M. (2012). Study of mixed Ca–Zn hydroxyapatite surface modified by lactic acid. Appl. Surf. Sci..

[27] Sakamoto K., Yamaguchi S., Nakahira A., Kaneno M., Okazaki M., Ichihara J., Tsunawaki Y., Elliott J.C. (2002). Shape-controlled synthesis of hydroxyapatite from α-tricalcium bis(orthophosphate) in organic-aqueous binary systems. J. Mater. Sci..

[28] Rong G.Z., Deng M.X., Deng C., Tang Z.H., Piao L.H., Chen X.S., Jing X.B. (2003). Synthesis of poly(ε-caprolactone)-*b*-poly(γ-benzyl-l-glutamic acid) block copolymer using amino organic calcium catalyst. Biomacromolecules.

[29] Lim J.S., Kim J.H. (2009). New application of poly(butylene succinate) (PBS) based ionomer as biopolymer: A role of ion group for hydroxyapatite (HAp) crystal formation. J. Mater. Sci..

[30] Pan Y.S., Xiong D.S., Chen X.L. (2007). Mechanical properties of nanohydroxyapatite reinforced poly(vinyl alcohol) gel composites as biomaterial. J. Mater. Sci..

[31] Kandori K., Oda S., Fukusumi M., Marisada Y. (2009). Synthesis of positively charged calcium hydroxyapatite nano-crystals and their adsorption behavior of proteins. Colloids Surf. B Biointerfaces.

[32] Mori H., Iwata M., Ito S., Endo T. (2007). Ring-opening polymerization of γ-benzyl-l-glutamate-N-carboxyanhydride in ionic liquids. Polymer.

